# Transcription factor Snai1-1 induces osteosarcoma invasion and metastasis by inhibiting E-cadherin expression

**DOI:** 10.3892/ol.2014.2079

**Published:** 2014-04-22

**Authors:** HUIGUANG YANG, YUNQING ZHANG, ZHENGMING ZHOU, XUEFENG JIANG, AIDONG SHEN

**Affiliations:** Department of Orthopaedics, The Affiliated Jiangyin Hospital of Southeast University Medical College, Jiangyin, Jiangsu 214400, P.R. China

**Keywords:** Snail-1, E-cadherin, epithelial-mesenchymal transition, osteosarcoma

## Abstract

Osteosarcoma (OS) is a type of primary malignant bone tumor with a high propensity for local recurrence and distant metastasis. A previous study showed Snail-1 is highly expressed in OS cells. The present study aimed to investigate the association between the transcription factor Snai1 and E-cadherin in OS. SaOS_2_ OS cells were transfected either with a plasmid expressing short hairpin RNA (shRNA) specific for the Snai1-1 gene (SaOS_2_-shRNA) or a negative control plasmid (SaOS_2_-Mock). The expression levels of E-cadherin and Snai1-1 in the transfected and control cells were determined by quantitative polymerase chain reaction and western blot analysis. In addition, the study was extended to evaluate the migratory and invasive properties of the cells through a Transwell experiment. The results show that E-cadherin was expressed at a high level in the SaOS_2_-shRNA cells, which were much less migratory and invasive than the control cells. Overexpression of Snai1-1 in OS is associated with tumor progression, possibly through the suppression of E-cadherin expression and induction of the process of epithelial-mesenchymal transition, which contributes to the proceeding invasion and metastasis of OS cells.

## Introduction

Osteosarcoma (OS) is the most common type of primary malignancy of bone. Despite intensive chemotherapy and adequate surgical resection, ~30–50% of patients succumb to OS, mainly due to distant metastasis to the lung (1,2). Snail-1 is a zinc-finger transcription factor expressed in migratory processes during embryonic development that has been implicated in cancer (3,4). A previous study showed that Snail-1 is highly expressed in OS cells, and is associated with the migratory and invasive properties of OS cells (5).

Studies have shown that Snail-1 upregulation in epithelial cells induces the expression of E-cadherin. Snail-1 contributes to the maintenance of the adhesive and polarized phenotype of epithelial cells where it is mainly expressed (6). If E-cadherin expression is downregulated, the epithelial cells acquire a fibroblastoid morphotype accompanied by the acquisition of invasive and migratory properties (7–9). This event is critical for the invasion and metastasis of carcinoma cells (10,1). Therefore, the loss of E-cadherin expression may be considered as an indicator of poor clinical prognosis and it is important to identify the molecular mechanism that regulates the expression of E-cadherin. A study has identified that E-cadherin is regulated by transcriptional factors such as Snail-1 (12). In the present study, whether Snail-1 regulates E-cadherin expression in OS cells was investigated and the association between E-cadherin expression and the migratory and invasive properties of the cells was explored

## Materials and methods

### Cell line and culture

The human SaOS_2_ OS cell line was purchased from the China Center for Type Culture Collection (Wuhan, China). The cells were cultured in McCoy’s 5A medium (Hyclone, Logan, UT, USA) supplemented with 10% fetal bovine serum (FBS; Hangzhou Sijiqing Biological Engineering Materials Co. Ltd., Hangzhou, China) and antibiotics (100 U/ml penicillin and 100 μg/ml streptomycin) in humidified air with a 5% CO_2_ atmosphere at 37°C (Thermo Direct Heat CO_2_ incubator; Thermo Fisher Scientific, Waltham, MA, USA). The study was approved by the ethics committee of the Affiliated Jangyin People’s Hospital of Southeast University Medical College (Jiangyin, Jiangsu).

### Transfection

The SaOS_2_ cells were plated in 100-mm dishes and transfected at 50–80% confluence with an expression vector for short hairpin RNA (shRNA) targeting Snail-1 or with a control vector, using the liposome-mediated transfection method (5). To establish cells in which Snail-1 expression was stably suppressed and mock-transfected cells, the SaOS_2_ cells were transfected with a plasmid (pcDNA3.1/shSnail-1 or pcDNA3.1/GAPDH, respectively; Introgen Therapeutics Inc., Austin, TX, USA) for two days. The cells were then trypsinized and plated at a low density. The stable clones were selected by maintaining the cells in medium containing the antibiotic G418 (Nanjing KeyGen Biotech Co., Ltd., Nanjing, China).

### Animal

BALB/c nude mice (between six and seven weeks old) were obtained from the Shanghai SLRC Laboratory Animal Co., Ltd. (license no. SCXK [hu] 2007–0005; Shanghai, China). The mice were bred and housed in a pathogen free, temperature-controlled and air-conditioned environment with a 10/14 h light/dark cycle. Two groups of these immunodeficient mice were subcutaneously injected with either mock- or Snail-1 shRNA-transfected cells [1×10^6^ cells/200 μl phosphate-buffered saline (PBS)]. Tumor growth was measured with a caliper two or three times every two days. Tumor volume was calculated using the following formula: Volume = (L × W^2^)/2, where L is the tumor length and W is the tumor width (both in millimeters) and L>W. After 17 days the mice were sacrificed in accordance with the guidelines for the welfare of animals in experimental neoplasia and the tissues were stored at −80°C.

### Cell viability assay

The viability of the cells was determined by the Cell Counting kit-8 (CCK-8; Nanjing KeyGen Biotech. Co. Ltd., Nanjing, China) assay. The cells (1×10^4^/well) were plated in 96-well plates in 200 μl medium per well. At different time points, the CCK-8 solution was added and the cells were cultured for 4 h. The absorbance at 570 nm was measured with a microplate reader (Sunrise; Tecan, Männedorf, Switzerland), using wells without cells as blanks and using untreated cells as the negative control. Cell death was calculated as a percentage of inhibition using the following formula: inhibition (%) = (1 - mean experimental absorbance/mean control absorbance) × 100.

### Apoptosis detection

Apoptosis detection of the cells was performed using a TRITC Staining Apoptosis Detection kit (Nanjing KeyGen Biotech. Co. Ltd.) and flow cytometry (BD Biosciences, San Jose, CA, USA). Briefly, the cells were trypsinized, washed with PBS, centrifuged and fixed for 20 min at 15–25°C (fixation solution: 4% paraformaldehyde in PBS buffer, pH 7.4, freshly prepared). The cells were washed for 30 min with PBS, and then were incubated with blocking solution for 10 min at 15–25°C (the blocking solution contained 3% H_2_O_2_ in methanol). Subsequently, the cells were incubated in permeabilization solution for 2 min on ice (2–8°C; the permeabilization solution contained 0.1% Triton X-100 and 0.1% sodium citrate, freshly prepared). Terminal deoxynucleotidyl transferase (TdT) dUTP nick end labeling (TUNEL) reaction mixture (50 μl; Nanjing KeyGen Biotech Co., Ltd.) was added to the samples and the samples were incubated for 60 min at 37°C in a wet and dark atmosphere (TUNEL reaction mixture contained 45 μl equilibration buffer, 1 μl TRITC-5-dUTP and 4 μl TdT, freshly prepared), then resuspended in 500 μl 4′,6-diamidino-2-phenylindole (DAPI)/RNase buffer (Nanjing KeyGen Biotech Co., Ltd.) and incubated for 30 min at 37°C. The samples were assayed by fluorescence microscopy using an excitation wavelength of 543 nm and emission wavelength of 571 nm (green) within 1 h.

### Matrigel invasion assay

A modified Boyden chamber (Neuro Probe Inc., Gaithersburg, MD, USA) was used. The pore size of the polycarbonate filters was 8.0 mm. The bottom chamber of the Transwell chamber was filled with 30 ml McCoy’s 5A medium containing 10% FBS. The cells were then suspended at a density of 1×10^5^ cells/ml in 500 ml McCoy’s 5A medium supplemented with 0.5% FBS, and 1,25(OH)_2_-D_3_ with a concentration of 10^−6^ M was added to the 8-mm porous BD BioCoat Matrigel chamber inserts (BD Biosciences, San Jose, CA, USA). Subsequently, the inserts were placed in the wells which were filled with 0.7 ml medium supplemented with 10% fetal calf serum as a chemoattractant. After two days of incubation, the upper side of the filter was scraped with a cotton tip to eliminate cells that had not migrated through it. The invasive ability of the cells was determined by counting the cells that had migrated to the lower side of the filter with a microscope. The experiments were performed in triplicate and ≥10 fields were counted in each experiment.

### Western blot analysis

The proteins were extracted from the SaOS_2_ cells in lysis buffer and then separated by SDS-PAGE and electrophoretically transferred onto polyvinylidene difluoride membranes. The membranes were probed with primary antibodies overnight at 4°C, and then incubated with the secondary antibodies. The images of the western blot products were collected and analyzed with Quantity One V4.31 software (Bio-Rad, Hercules, CA, USA). The primary Snail-1 and E-cadherin polyclonal antibodies were purchased from Abnova (Taiwan, China). Goat anti-mouse HRP-conjugated secondary antibody was obtained from Santa Cruz Biotechnology, Inc. (Santa Cruz, CA, USA) and goat anti-rabbit HRP-conjugated secondary antibody was purchased from Amersham Pharmacia Biotech (Piscataway, NJ, USA).

### Quantitative polymerase chain reaction (qPCR)

Total RNA was isolated from the SaOS_2_ cells using TRIzol reagent (Invitrogen Life Technologies, Carlsbad, CA, USA) according to the manufacturer’s instructions. The reverse transcription reactions were conducted with a Transcriptor First Strand cDNA Synthesis kit (Roche, Indianapolis, IN, USA). The PCR primers were designed by Premier Primer software, version 5.0 (Premier Biosoft, Palo Alto, CA, USA). qPCR with SYBR Green PCR Master mix (Applied Biosystems, Foster City, CA, USA) was performed using an ABI Prism 7500 Sequence Detection system (Applied Biosystems). The fluorescent signals were collected during the extension phase, the Ct values of the sample were calculated and the transcript levels were analyzed by the 2^−ΔΔCt^ method.

### Data analysis

Statistical comparisons were performed with the software package SPSS, version 13.0 (SPSS, Inc., Chicago, IL, USA) using Student’s t-test for paired observations or one-way analysis of variance with Student-Newman-Keuls, least significant difference and Dunnett’s methods. All data are presented as the mean ± standard deviation (SD). P<0.05 was considered to indicate a statistically significant difference. The mean values and SD were calculated for the experiments conducted in triplicate.

## Results

### Inhibition of Snail-1 increases the expression levels of E-cadherin in SaOS_2_ cells

**Figure 1:**
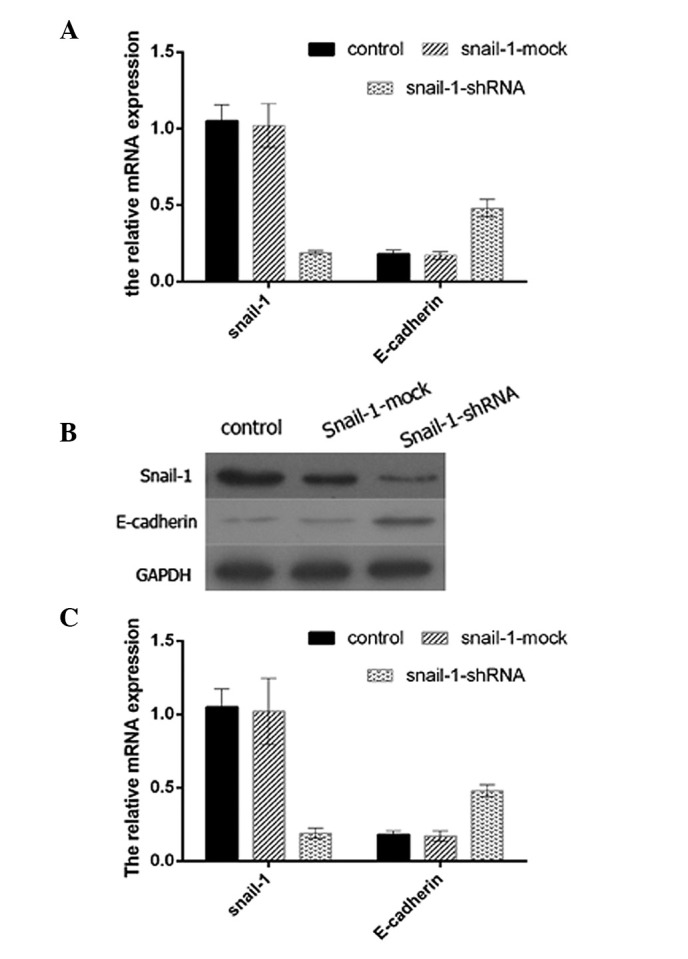
E-cadherin and Snail-1 expression levels following inhibition of Snail-1 in SaOS_2_ cells analyzed by western blotting and qPCR. GAPDH was used as a loading control. The relative (A and B) protein and (C) mRNA expression levels of Snail-1 and E-cadherin. The results are presented as the mean ± SD. The experiments were performed in triplicate.

To determine the association between Snail-1 and E-cadherin, shRNA targeting Snail-1 was successfully transfected into SaOS_2_ cells and the expression levels of Snail-1 and E-cadherin in the cells were detected by western blot analysis. To confirm the efficacy of Snail-1 shRNA, qPCR and western blot analysis were performed (data not shown). The expression levels of E-cadherin in the SaOS_2_ cells transfected with the Snail-1-shRNA vector were significantly higher than those in the SaOS_2_ cells with no treatment (control group) or infected with the negative control shRNA (shRNA-Mock) (Fig. 1). Therefore, inhibition of Snail-1 expression in SaOS_2_ cells resulted in higher levels of E-cadherin protein expression.

### Proliferation of SaOS_2_ cells and Snail-1-shRNA-transfected SaOS_2_ cells

**Figure 2:**
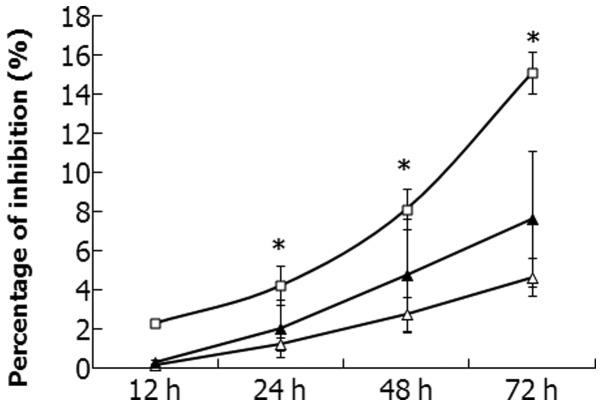
Comparison of the levels of growth inhibition of SaOS_2_ cells after different time periods. Growth inhibition was determined by the CCK-8 assay. (^□^shRNA-Snail-1 group, ^▲^shRNA-Mock group and ^△^control group) ^*^P<0.05 vs. control group. CCK-8, cell counting kit-8.

CCK-8 assays were performed to investigate the proliferation of the SaOS_2_ cells transfected with shRNA-Mock and shRNA-Snail-1. The growth inhibition of all cells increased in a time-dependent manner (Fig. 2). The results suggest that the growth of the SaOS_2_ cells was inhibited significantly when Snail-1 expression was inhibited (P<0.05).

### Apoptosis differs between normal and sh-Snail-1-transfected SaOS_2_ cells

**Figure 3:**
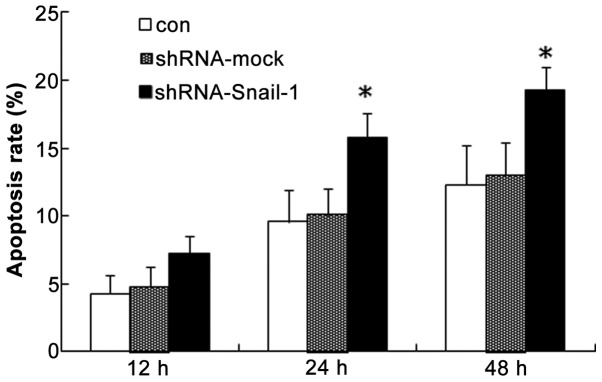
Comparison of the apoptotic rate of SaOS_2_ cells following transfection with shRNA-Snail-1 at different time periods. ^*^P<0.05, indicating that the rate of apoptosis was significantly higher than that of the control (con) and shRNA-mock groups.

A quantitative analysis of the fluorescent signals of the cells was performed by fluorescence-activated cell sorting. As shown in Fig. 3, the percentage of TRITC-positive SaOS_2_ cells was significantly increased from 12.3% in the control group to 20.3% in the SaOS_2_ cells transfected with shRNA-Snail-1 after treatment for 48 h (P<0.05). These data indicate that the rate of apoptosis of the SaOS_2_ cells increased when Snail-1 expression was inhibited.

### Change of the invasive ability of SaOS_2_ cells following transfection with shRNA-Snail-1

**Figure 4:**
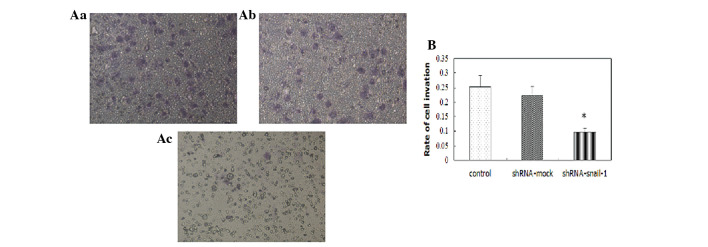
Invasiveness of SaOS_2_ cells. Cell invasion 48 h after treatment was evaluated in the Matrigel invasion assay. (A) Representative microscopic images of (a) untreated control cells, (b) cells transfected with shRNA-mock and (c) cells transfected with shRNA-Snail-1 after treatment (x200). (B) Comparison of the rate of cell invasion. Data are expressed as the percentage change (mean ± SD) and represent three independent experiments. ^*^P<0.05 vs. control.

A significant difference in the number of invading cells was observed between the SaOS_2_ cells transfected with shRNA-mock and those transfected with shRNA-Snail-1 in the migration assay. Furthermore, the invasion rate of the SaOS_2_ cells transfected with shRNA-Snail-1 was significantly lower than that of the control SaOS_2_ cells (P<0.05) in the Matrigel invasion assay (Fig. 4).

### Tumor growth in vivo

**Figure 5:**
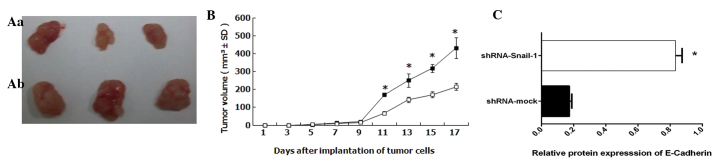
Growth of SaOS_2_ cells xenografted into BALB/c nude mice. The SaOS_2_ cells were implanted subcutaneously into the flanks of the mice. (A) Photograph of the SaOS_2_ tumors from the mice at day 17 after implantation in the (a) shRNA-mock and (b) shRNA-Snail-1 groups. (B) The tumor volume following implantation. White squares indicate the shRNA-mock group and black squares indicate the shRNA-Snail-1 group. Data are presented as the mean ± SEM (n=5 in each group). (C) Levels of E-cadherin detected in the tumor tissues. ^*^P<0.05 vs. shRNA-mock group.

Tumor growth was attenuated in the animals injected with cells transfected with shRNA-mock cells than in those injected with cells transfected with shRNA-Snail-1 (Fig. 5). The tumors generated by the cells transfected with shRNA-Snail-1 were less differentiated with much lower E-cadherin expression levels than those observed in shRNA-mock-transfected cells.

## Discussion

OS is the most common type of primary malignant tumor of bone in children and young adolescents (13,14). With the use of chemotherapeutics in combination with aggressive surgery, the long-term survival rate of patients has improved (15). However, a number of patients have metastases at initial diagnosis, which is common to the lung (16). Therefore, to more effectively control this disease and improve the patient survival rate, it is important to elucidate the molecular mechanism of human sarcomagenesis and to develop novel treatment options for OS.

Epithelial-mesenchymal transition (EMT) has been widely studied for its role in early development and cancer metastasis. EMT results in the transformation of a differentiated epithelial cell to a mesenchymal cell with stem-like properties and is characterized by loss of cell-to-cell adhesion, specifically through the dismantling of adherens, tight and gap junctions, as well as loss of cell polarity and increased motility (17,18). In embryogenesis, EMT functions by promoting the migration of mesenchymal cells during gastrulation and neural crest cell migration, then later during tissue remodeling and organogenesis, ultimately contributing to the development of differentiated tissues with specific phenotypes (3,19,20). In cancer progression, EMT appears to be at least partially responsible for the invasive nature of tumor cells and it facilitates metastasis by converting a non-motile cancerous epithelial cell into a motile mesenchymal cell capable of disseminating from the tumor mass and entering the circulatory or lymphatic system (21). In a number of studies, EMT has been associated with the progression of numerous types of cancer (22–24), but few of these studies concerned OS. The loss of E-cadherin is the hallmark of EMT in cancer development (25,26). The present study showed that with decreased E-cadherin expression levels, the malignant biology behavior of cells was repressed, indicating that EMT was also associated with the progress of OS (Figs. 2–4).

Snail is the first identified and most important transcriptional repressor of E-cadherin (27,28). It functions as a suppressor of the transcription of shotgun (an E-cadherin homolog) to control embryogenesis in Drosophila (3,29). Snail also plays a fundamental role in EMT by suppressing E-cadherin expression in mammalian cells (30,31). Overexpression of Snail has been identified in epithelial and endothelial cells of invasive breast cancer but the overexpression of Snail is not detected in normal breast cells. The expression of Snail in breast carcinomas is associated with metastasis, tumor recurrence and poor prognosis (32–34). In a previous study, it was shown that Snail-1 was overexpressed in SaOS_2_ cells (5). Snail also downregulates the expression levels of other epithelial molecules, including claudins, occludins and Muc1, and induces the expression of genes associated with a mesenchymal and invasive phenotype, such as fibronectin and matrix metallopeptidase-9. The Snail family of zinc-finger transcription factors consists of Snail-1 (Snail), Snail-2 (Slug) and Snail-3 (Smuc) (3). In the present study Snail-1 was focused on and it was hypothesized that Snail-1 is able to regulate EMT in OS through E-cadherin.

To test this hypothesis, SaOS_2_ cells were treated with shRNA-Snail-1. As a result, following inhibition of Snail-1, the expression levels of E-cadherin decreased and the cells were less able to grow and invade and easily underwent apoptosis. E-cadherin is involved in EMT associated with carcinogenesis (25,27,28). Loss of E-cadherin has been causally associated with the transition of adenoma to carcinoma and the acquisition of migration capacity. Therefore, according data in the present study (Fig. 1), we considered that inhibition of the expression of Snail-1 prevents EMT and induces E-cadherin expression in OS. The results of the present study predict that patients with OS with high levels of Snail-1 and low levels of E-cadherin have a poor prognosis for the progression of OS. The expression levels of Snail-1 and E-cadherin may be used as indicators of the progression of OS. The monitoring of tumor overexpression of Snail-1 and E-cadherin by reverse transcription-PCR analysis of the RNA present in the serum/plasma may be useful as a non-invasive method for selecting suitable patients for therapy.

In conclusion, the present study indicates that Snail-1 is a regulator of E-cadherin and inhibition of Snail-1 represses EMT in OS, therefore Snail-1 has potential as a target of OS therapy.
